# Analysis of Factors Affecting Cranial Nerve Function of Patients With Vascular Mild Cognitive Impairment Through Functional Magnetic Resonance Imaging Under Artificial Intelligence Environment

**DOI:** 10.3389/fpubh.2021.803659

**Published:** 2022-03-25

**Authors:** Lifang Zhang, Yanran Li, Lin Bian, Qingrong Luo, Xiaoxi Zhang, Bing Zhao

**Affiliations:** ^1^Department of Neurology, Changzhi People's Hospital, Changzhi Medical College, Changzhi, China; ^2^Department of Mental Health, Changzhi Medical College, Changzhi, China; ^3^Department of Radiology, First Affiliated Hospital of Xinjiang Medical University, Urumqi, China

**Keywords:** artificial intelligence, vascular mild cognitive impairment, computer functional magnetic resonance imaging, neuropsychological assessment, whole brain regional homogeneity

## Abstract

The study aimed to explore the risk factors of effects of patients with vascular mild cognitive impairment (VaMCI) through functional magnetic resonance imaging (fMRI). In this study, 62 patients were selected from the department of neurology, admitted to Changzhi People's Hospital from October 1, 2018 to February 1, 2020. Patients with VaMCI were defined as the VaMCI group according to Clinical Dementia Rating (CDR), and subjects with normal cognitive function were defined as the normal control (NC) group. All patients underwent fMRI to identify the amplitude low-frequency fluctuation (ALFF) and regional homogeneity (ReHo) values, and to analyze their association with VaMCI. The results showed that the VaMCI group had lower scores for Mini-mental State Examination (MMSE), Montreal Cognitive Assessment (MoCA), and their subitems (visual space and execution, recall, attention and computation, and language ability) than NC group, with statistical differences (*P* < 0.05). In VaMCI group, the brain regions with increased ALFF values were the left temporal lobe, left parietal lobe, right temporal lobe, right parietal lobe, and posterior cingulate gyrus. Of them, the left parietal lobe and right temporal lobe were negatively correlated with the recall score on MMSE scale (*r* = −0.216, *r* = −0.132, *P* < 0.01). In VaMCI group, the brain regions with decreased ReHo values were the left temporal lobe, occipital lobe, and left middle temporal gyrus. Of them, the left temporal lobe and occipital lobe were positively correlated with MoCA score (*r* = 0.473, *r* = 0.848, *P* < 0.01). In conclusion, VaMCI patients have cognitive impairment and abnormally increased spontaneous brain activity, especially in the left parietal lobe and the right temporal lobe. At rest, VaMCI patients show decreased whole-brain ReHo in the left medial temporal lobe and occipital lobe. Hypertension is a high-risk factor for cognitive impairment in VaMCI patients. The study can provide a theoretical basis for early diagnosis of VaMCI.

## Introduction

Cerebrovascular diseases are a main factor causing vascular cognitive impairment (VCI) (1, 2). With the population aging, the incidence of VCI increases significantly, second only to Alzheimer's disease (3–5). Similar to other cognitive diseases, VCI is characterized by gait, emotion, behavior, and urination disorders (6, 7). Cognitive impairment in an early stage is often overlooked, while it is difficult to treat once it develops to the late stage, such as dementia (8). Vascular mild cognitive impairment (VaMCI), as a prodromal stage of VCI, is closely related to executive dysfunction (9, 10), so early detection of VaMCI is particularly important.

Currently, the clinical diagnosis of VaMCI mainly relies on neurological function evaluation and imaging examination. Of many diagnostic methods, artificial intelligence-based imaging is of great significance for early diagnosis of VaMCI (11–14). With the continuous improvement of modern medical technology, artificial intelligence (AI) technology is gradually introduced into fMRI and plays an important role. AI can mine effective imaging information from big data, providing convenience for doctors in the diagnosis and treatment of cerebrovascular diseases (15–18). The fMRI imaging technology is now widely used to explore the mechanism of cerebrovascular cognitive function by detecting the activity of brain neurons. When different activities occur, both vascular cognitive function and neuronal activity have corresponding changes (19). Artificial intelligence-based fMRI technology features non-invasiveness, repeatable and high spatial resolution. It is an important means to study the neural mechanism of VCI patients (20–23). However, there are few studies to use fMRI technology to diagnose VaMCI, and the related research is still in the exploratory stage at present.

In this study, case data and fMRI data of patients in the department of neurology and the department of physical examination, admitted to Changzhi People's Hospital from October 1, 2018 to February 1, 2020, were collected, to identify the ALFF and ReHo value, and to analyze their association with VaMCI, expected to provide a basis for the early diagnosis of cerebrovascular diseases.

## Materials and Methodology

In this study, the subjects underwent fMRI to identify the ALFF and ReHo values, and to analyze their association with VaMCI, aiming to explore the risk factors of VaMCI by fMRI combined with neuropsychology under artificial intelligence.

### Basic Information of Research Subjects

Case data and fMRI data of patients in the department of neurology and the department of physical examination, admitted to Changzhi People's Hospital from October 1, 2018 to February 1, 2020, were collected. Thirty two patients were in the VaMCI group according to the diagnostic criteria of CDR and the Guidelines for the Diagnosis and Treatment of Vascular Cognitive Impairment in China in 2019 (24). A total of 30 healthy patients without memory loss were in the NC group. The subjects in the NC group had no other related disorders that might affect cognitive function.

Inclusion criteria: 1. patients with complete case history and fMRI data; 2. acute cerebrovascular diseases did not occur within at least 1 year; 3. patients diagnosed with VCI as per the criteria of *the Guidelines for the Diagnosis and Treatment of Vascular Cognitive Disorders in China* in 2019; 4. there were cerebrovascular risk factors, and no evidence of cerebrovascular diseases such as hydrocephalus, intracerebral hemorrhage, lacunar cerebral infarction, and leukodystrophy from fMRI; and 5. other intracranial lesions were excluded, such as: tumor, trauma, infection or surgery.

Exclusion criteria: 1. Intracranial vascular color doppler ultrasound, head fMRI or head CT angiography showed intracranial vascular stenosis of more than 50%; 2. patients with abnormal neurological diseases such as Parkinson's disease, brain tumor, and encephalitis; 3. patients unable to complete the examination due to moderate aphasia, depression, or mental disorder; 4. patients with genetic microvascular diseases; and 5. patients with a history of major depression, drug, alcohol, or drug dependence.

### Neurological Function Evaluation

The environment was quiet where the patient was tested. The test scales include MMSE, MoCA, Activities of Daily Living (ADL), and CDR (25), factoring into subitems such as memory, numeral symbol conversion, attention and computational power, visual space and executive function, recall power, orientation, language ability, and abstract ability. Neurological function evaluation was performed by neurologists with years of experience.

### MRI Examination

MRI scanner used the Siemens verio 3.0T computer. The patient was in a supine position with his head in his hands. The patient should breath peacefully but not to think or fall asleep. For patients unable to cooperate in the MRI examination, chloral hydrate can be taken orally for sedation. The patient was scanned for conventional horizontal position, axial T2WI, vector T1WI, coronary T2, and fluid attenuated inversion recovery (FLAIR) images, and scanning parameters are set as follows:

- Conventional scanning: scanning voltage of 120 kV, current of 150mA, with T1WI repetition time (TR) of 1800 ms, time of echo (TE) of 25 ms, T2WI (TR/TE = 2800/75 ms), FLAIR (TR/TE = 7500/120 ms), matrix 525 × 525, scanning layer thickness of 4 mm, and layer spacing of 5 mm.
- fMRI scanning: TR/TE = 1800/30 ms, matrix 70 × 70, scanning layer thickness of 3 mm, layer distance of 1 mm. there are a total of 250 time points in 10 min.

### Pretreatment and Analysis of fMRI Image Data

The MRI data are processed by Statistic parameter Mapping (SPM8) software of Matlab platform, and the specific steps are shown below (26).

- Data conversion: SPM8 software converts the original data Dicom into NII format.
- Time correction: all images are obtained at the same time to eliminate the influence of time difference on the data.
- Head motion correction: during the scan, the patient's breathing and heartbeat will have certain influence on the head, so it is necessary to keep head motion within a maximum shift of 3 mm.
- Space standardization: due to brain morphology differences, space standardization is required. The volume size is set as 3 × 3 × 3 mm for resampling.
- Spatial smoothing: spatial processing with 4 × 4 × 4 mm full width at half maximum (FWHM) is performed to suppress high frequency signal and image noise.
- De-linear drift and low frequency filtering: in order to eliminate baseline drift, it is necessary to remove linear drift, and the frequency band of 0.01~ 0.09 hz is used for low-frequency filtering to remove high-frequency interference.
- Calculation of ALFF: the ALFF value indicates the activity of voxel in the brain, which is positively correlated with spontaneous brain activity. DPARSF software is used to calculate the average amplitude value between 0.01 and 0.09 Hz.

### Calculation of ReHo

A high ReHo value indicates consistent local neuron signal activity and a low value indicates decreased consistency. The ReHo value of each voxel is obtained by computer analysis, and the ReHo values of all brain regions can form a complete ReHo diagram.

### Statistical Analysis

SPSS13.0 statistical software is used to analyze and process all experimental data. The count experimental data are expressed as a percentage (%) and the measurement data are expressed as mean ± standard deviation ( ± s). The χ*2* test is used for the gender and education background analyses of the patients. *T-*test is used for age comparison between the two groups. The fMRI data are processed by DPARSF and SPM8 software, and the differences in ALFF and ReHo are analyzed by *t*-test. SPSS Pearson software is employed to analyze the correlation between ALFF, ReHo with MMSE, MoCA. *P* < 0.05 indicates statistically significant differences.

## Results

### Basic Information

**Figure 1:**
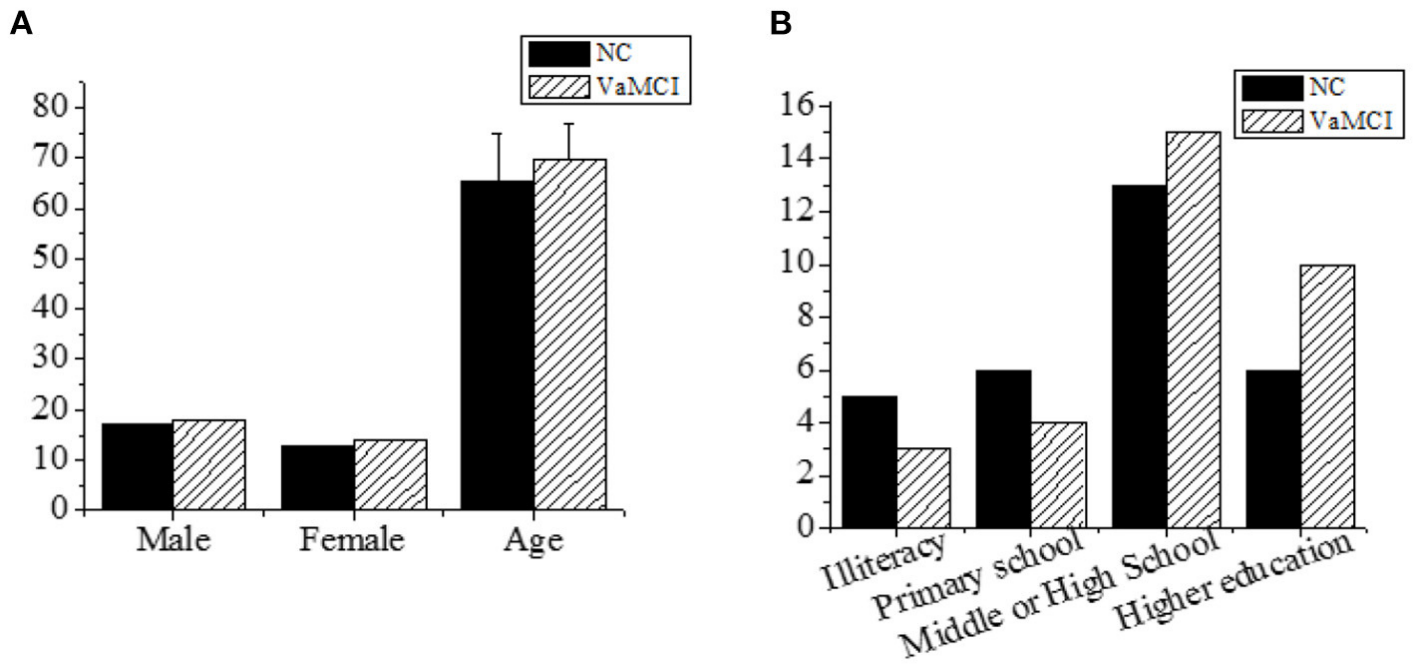
The basic information. **(A)** The age and gender; **(B)** the education background.

Figure 1 showed the gender and age of patients in NC group and VaMCI group. In the NC group, there were 17 males (56.7%) and 13 females (43.3%). In the VaMCI group, there were 18 males (56.3%) and 14 females (43.7%). The χ^2^-test results showed no significant differences in gender (*P* > 0.05). The subjects in NC group were aged between 45–85 years old and averaged 65.3 ± 9.38; the patients in VaMCI group were aged between 50–88 years old, and averaged 69.54 ± 7.23. There was no significant difference in age after *t-*test (*P* > 0.05). The basic information was comparable in gender and age (*P* > 0.05). In terms of the education background of the two groups, 5 cases of illiteracy in NC group accounted for 16.7%, 6 cases of primary school accounted for 20%, 13 cases of junior high school accounted for 43.3%, and 6 cases of higher education accounted for 20%. In VaMCI group, 3 cases of illiteracy accounted for 9.4%, 4 cases of primary school accounted for 12.5%, 15 cases of junior high school accounted for 46.9%, and 10 cases of higher education accounted for 31.2%. The χ^2^-test showed no significant difference in educational background (*P* > 0.05).

### The Medical History and Family History

**Figure 2:**
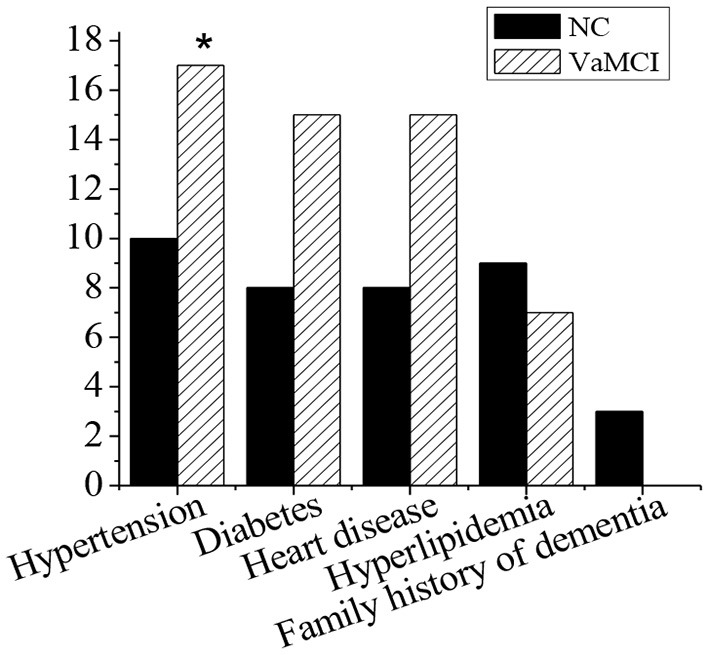
Medical history and family history. There was a statistically significant difference in hypertension between the VaMCI group and the control group (*P < 0.05).

Figure 2 showed the medical history and family history of patients in NC group and VaMCI group. In NC group (30 cases), 10 cases of hypertension accounted for 33.3%, 8 cases of diabetes accounted for 26.7%, 8 cases of heart disease accounted for 26.7%, 9 cases of hyperlipidemia accounted for 30%, and 3 cases with a family history of dementia accounted for 10%. In VaMCI group (32 cases), there were 17 cases with hypertension (53.1%), 15 patients with diabetes (46.9%), 15 patients with heart disease (46.9%), 7 patients with hyperlipidemia (21.9%), and 0 patients with a family history of dementia. The number of hypertension patients in VaMCI group was significantly higher than that in the control group (^*^P < 0.05).

### MMSE and Subitem Scores

**Figure 3:**
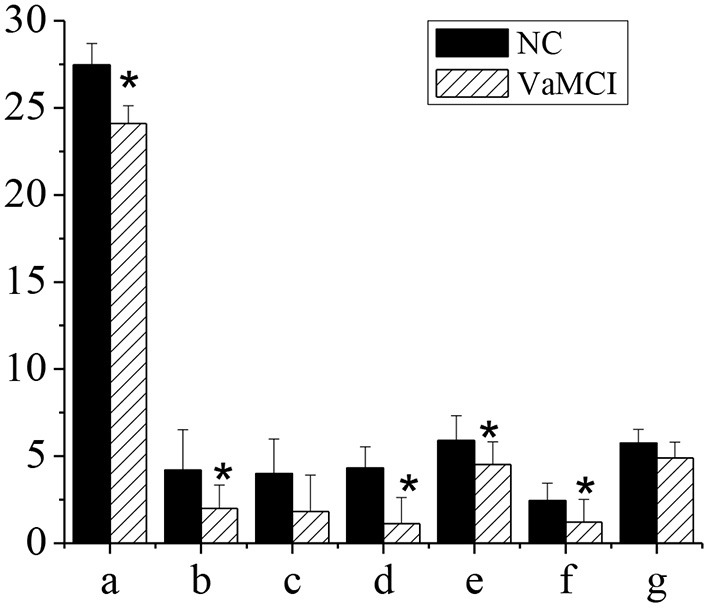
MMSE and subitem scales. The comparison between the NC group and the VaMCI group was statistically significant, *P < 0.05. a-MMSE; b-visual space and execution; c-memory; d-recall power; e-attention and computation; f-language ability; g-orientation.

Figure 3 showed the MMSE and subitem acores of patients in NC group and VaMCI group. The total MMSE scores of VaMCI group were (24.11 ± 1.01), the subitem scores were (2.01 ± 1.34) for spatial and executive ability, (1.12 ± 1.5) for recall ability, (4.52 ± 1.3) for attention and computational ability, and (1.22 ± 1.3) for language ability, all of which were lower vs. the NC group, where the total MMSE score was (27.46 ± 1.23), and the subitem scores were (4.2 ± 2.33) for spatial and executive ability, (4.33 ± 1.21) for recall power, (5.89 ± 1.44) for attention and computational ability, and (2.45 ± 0.99) for language ability (*P* < 0.05). There was no statistically significant difference in memory and orientation between the NC group and the VaMCI group (*P* > 0.05).

### MoCA and Subitem Scores

**Figure 4:**
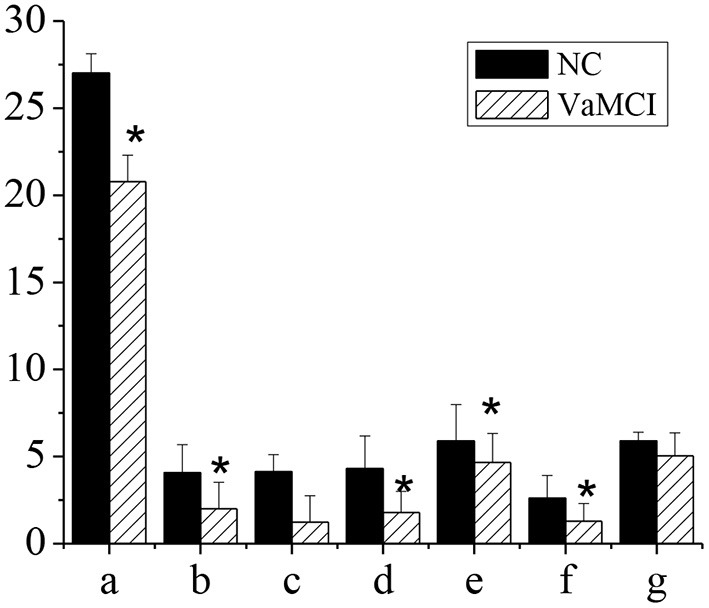
MoCA and subitem scores. The comparison between the NC group and the VaMCI group was statistically significant *P < 0.05. a-MMSE; b-visual space and execution; c-memory; d-recall power; e-attention and computation; f-language ability; g-orientation.

Figure 4 showed the MoCA and subitem scores of patients in NC group and VaMCI group. The total MoCA score of VaMCI group was (20.78 ± 1.52), the subitem scores were (2.01 ± 1.51) for spatial and executive ability, (1.79 ± 1.22) for recall power, (4.66 ± 1.67) for attention and computational ability, and (1.29 ± 1.01) for language ability, all of which were lower vs. the NC group, where the total MoCA score was (27.01 ± 1.12), and the subitem scores were (4.08 ± 1.61) for spatial and executive ability, (4.3 ± 1.89) for recall power, (5.89 ± 2.1) for attention and computational ability, and (2.61 ± 1.3) for language ability (*P* < 0.05). There was no statistically significant difference in memory and orientation between the NC group and the VaMCI group (*P* > 0.05).

### The fMRI Characteristics

**Figure 5:**
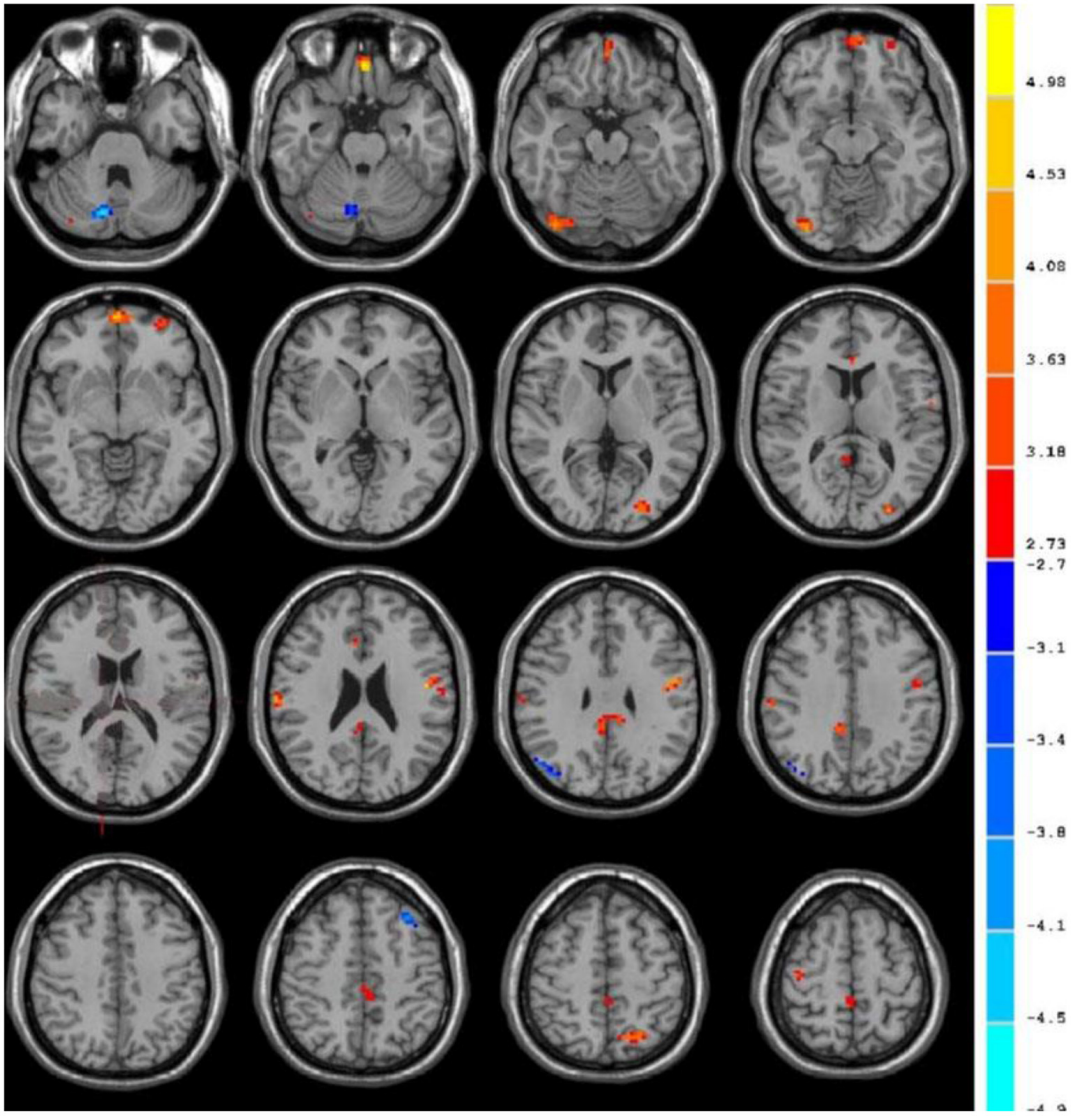
fMRI images to show ALFF. The blue area represented the brain areas with increased ALFF in VaMCI group, the orange area represented the brain areas negatively correlated with the scale score, and the color bar represented the size of T value.

Table 1 showed the ALFF value of each brain region in the VaMCI group. When the number of continuous volume pixel is greater than 50, the cluster is defined as a brain region, as shown in Figure 5. Compared with the NC group, there were 7 clusters with increased ALFF values in VaMCI group, which were located in the left temporal lobe, left parietal lobe, right temporal lobe, right parietal lobe, and posterior cingulate gyrus, with statistical significances (*P* < 0.05). No statistical differences were noted in brain areas of the left occipital lobe and the right occipital lobe (*P* > 0.05), and there was no brain area with abnormally decreased ALFF.

**Table 1:** Numerical analysis of ALFF in each brain region of VaMCI group.

**Brain area**	**MINI coordinate**			***T* value**	**Voxel**
	**x**	**y**	**z**		
Left temporal lobe	−46	−15	29	−2.98	76
Left occipital lobe	58	21	42	−3.67	42
Left parietal lobe	−18	30	17	+2.54	52
Right temporal lobe	−62	−18	40	−4.28	61
Right occipital lobe	47	71	27	−3.17	30
Right parietal lobe	−8	−46	59	+3.26	51
Posterior cingulate	11	−51	25	+3.54	65

*MNI, human brain region coordinates; T value, activation degree*.

### ReHo Values

**Figure 6:**
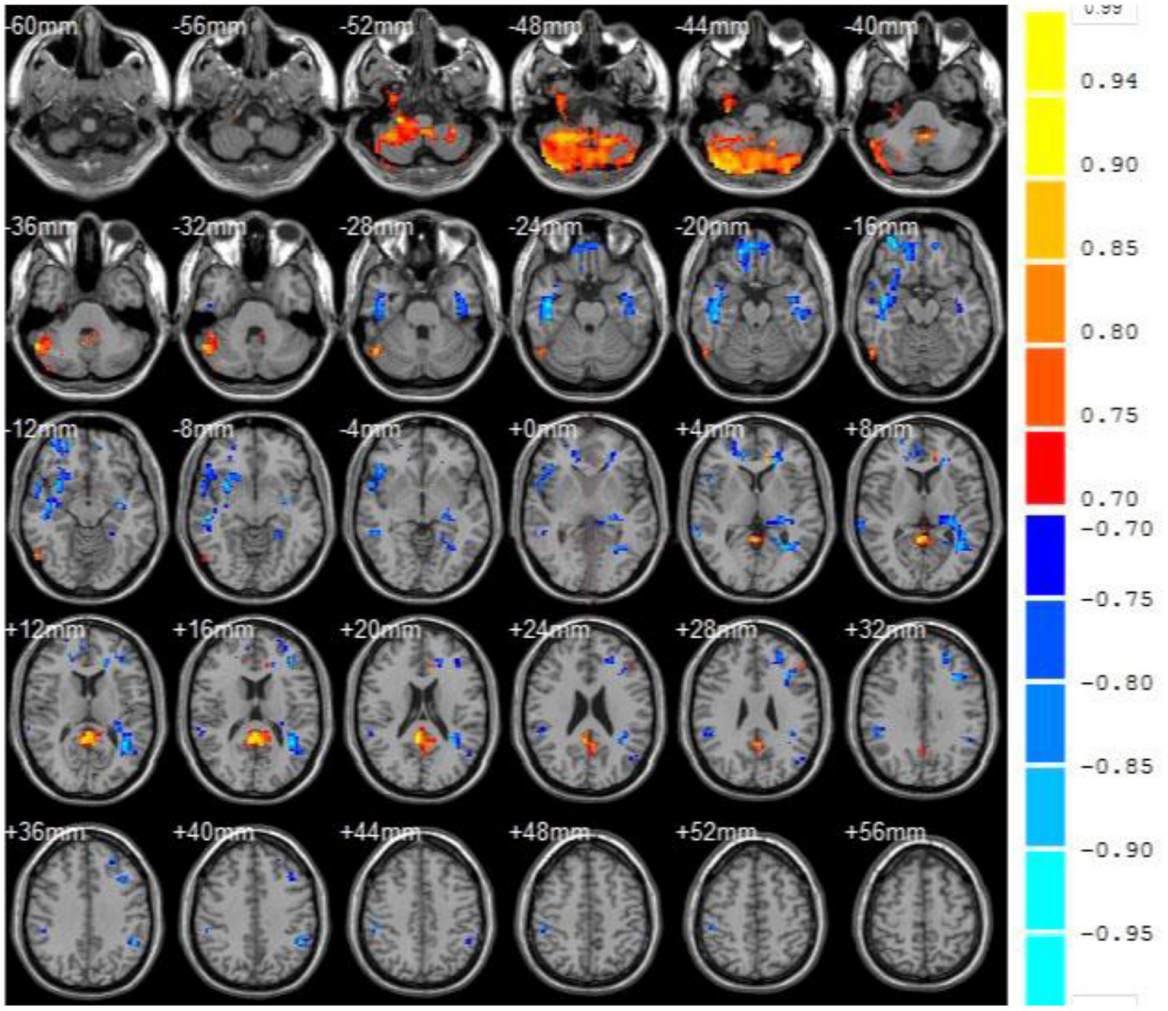
fMRI images to show ReHo. Compared with the NC group, the blue area represented the brain areas with decreased ReHo value in VaMCI group, the orange area represented the brain areas negatively correlated with the score, and the color bar represented the size of T value.

Also, the cluster is defined as a brain region when the number of continuous voxels is greater than 50, as shown in Figure 6. Table 2 showed the ReHo value of each brain area in VaMCI group. Compared with the NC group, there were 6 clusters with decreased ReHo values in VaMCI group, which were located in the left temporal lobe, occipital lobe, and left middle temporal gyrus, with statistical differences (*P* < 0.05). No statistical difference was noted in the left inferior temporal gyrus, left Para hippocampal gyrus, and insula (*P* > 0.05). There were 4 clusters with increased ReHo values, which were the left superior temporal gyrus, the right frontal lobe, the left frontal lobe, and the left inferior frontal gyrus (*P* < 0.05).

**Table 2:** Numerical analysis of ReHo in different brain regions of VaMCI group.

**Brain region**	**MINI coordinate**			***T* value**	**Voxel**
	**x**	**y**	**z**		
Left temporal lobe	71	−59	−24	+2.98	74
Occipital lobe	−43	−64	−11	−3.67	53
Left inferior temporal gyrus	−46	−43	−10	+2.54	36
Left middle temporal gyrus	56	−35	−6	+4.28	84
Left Para hippocampal gyrus	47	47	21	−3.17	48
Insular lobe	26	8	5	+3.26	26
Left superior temporal gyrus	−44	5	−4	−2.89	58
Right frontal lobe	−14	16	43	+3.75	84
Left frontal lobe	−17	25	−7	−3.12	69
Left inferior frontal gyrus	−37	2	19	−2.91	52

*MNI, human brain region coordinates; T value: activation degree*.

### Spontaneous Brain Activity and Cognitive Impairment in VaMCI Group

**Figure 7:**
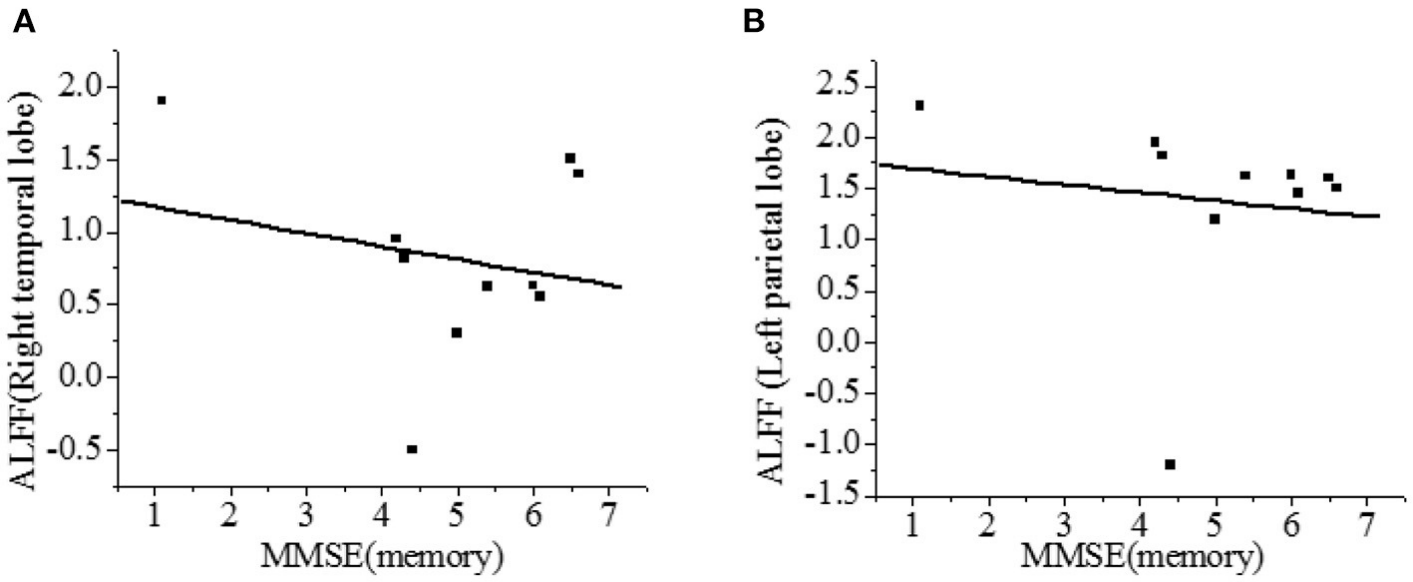
Correlation of ALFF with recall power of MMSE scale [**(A)**-right temporal lobe; **(B)**-left parietal lobe]. There was a significant difference, *P* < 0.01.

The correlation between the ALFF value and MMSE score was analyzed. As shown in Figure 7, the right temporal lobe and the left parietal lobe were negatively correlated with the recall power of MMSE scale (r = −0.216, r = −0.132, *P* < 0.01), and the difference was statistically significant.

### Correlation of ReHo With Cognitive Impairment

**Figure 8:**
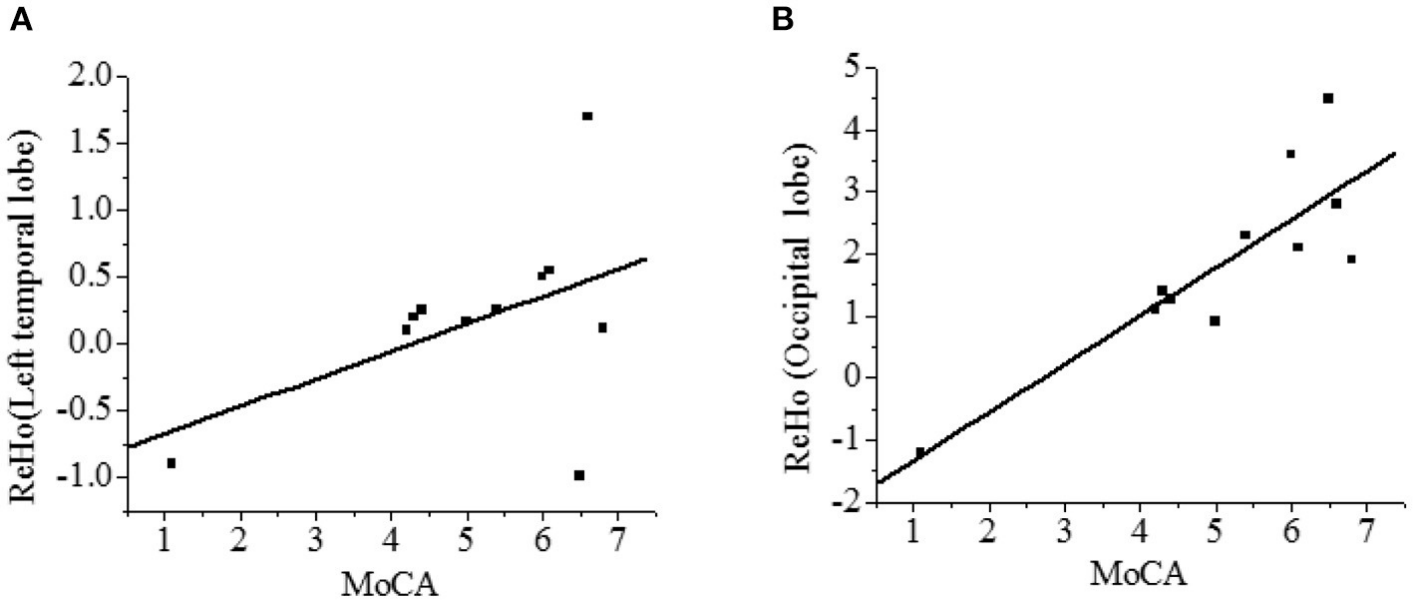
Correlation analysis between ReHo and MoCA score [**(A)**-left temporal lobe; **(B)**-occipital lobe]. There was a statistical significance, *P* < 0.01.

The correlation between the ReHo value and the MoCA score was analyzed. As shown in Figure 8, the ReHo in the left temporal lobe and occipital lobe was positively correlated with the recall power of the MoCA scale (*r* = 0.473, *r* = 0.848, *P* < 0.01), and the difference was statistically significant.

## Discussion

VCI has a serious impact on human health and life. VCI at an early stage is often ignored thanks to mild symptoms. When it develops into dementia, it is already too late and difficult to treat. Therefore, early detection of VaMCI is particularly important (27). In this study, case data and fMRI data based on AI technology of patients in department of neurology and department of physical examination, admitted to Changzhi People's Hospital hospital from October 1, 2018 to February 1, 2020 were collected. The total MMSE score of VaMCI group was (24.11 ± 1.01), the subitem scores were (2.01 ± 1.34) for spatial and executive ability, (1.12 ± 1.5) for recall ability, (4.52 ± 1.3) for attention and computational ability, and (1.22 ± 1.3) for language ability, all of which were lower vs. the NC group, where the total MMSE score was (27.46 ± 1.23), and the subitem scores were (4.2 ± 2.33) for spatial and executive ability, (4.33 ± 1.21) for recall power, (5.89 ± 1.44) for attention and computational ability, and (2.45 ± 0.99) for language ability (*P* < 0.05). There was no statistically significant difference in memory and orientation between the NC group and the VaMCI group (*P* > 0.05). It indicated that VaMCI patients had cognitive impairment, consistent with previous research (28). The total MoCA score of VaMCI group was (20.78 ± 1.52), the subitem scores were (2.01 ± 1.51) for spatial and executive ability, (1.79 ± 1.22) for recall power, (4.66 ± 1.67) for attention and computational ability, and (1.29 ± 1.01) for language ability, all of which were lower vs. the NC group, where the total MoCA score was (27.01 ± 1.12), and the subitem scores were (4.08 ± 1.61) for spatial and executive ability, (4.3 ± 1.89) for recall power, (5.89 ± 2.1) for attention and computational ability, and (2.61 ± 1.3) for language ability (*P* < 0.05). There was no statistically significant difference in memory and orientation between the NC group and the VaMCI group (*P* > 0.05). It indicated that memory and orientation in VaMCI group were less impaired than other subitems. Studies have confirmed that the damage of subfrontal cortical neurons in the brain was the main cause of VaMCI (29).

In this study, the ALFF value of fMRI image was used to study the local spontaneous brain activity of patients in VaMCI group. Compared with the NC group, there were 7 clusters with increased ALFF values in VaMCI group, which were located in the left temporal lobe, left parietal lobe, right temporal lobe, right parietal lobe, and posterior cingulate gyrus, with statistical significances (*P* < 0.05). No statistical differences were noted in brain areas of the left occipital lobe and the right occipital lobe (*P* > 0.05), and there was no brain area with abnormally decreased ALFF. It showed that the spontaneous activity was increased in the left temporal lobe, the left parietal lobe, the right temporal lobe, the right parietal lobe, and the posterior cingulate gyrus in the rest state of patients in VaMCI group. The right temporal lobe and the left parietal lobe were negatively correlated with recall power on MMSE scale (*r* = −0.216, *r* = −0.132, *P* < 0.01). The parietal lobe played an important role in controlling visual movement, eye movement, and attention (30). The analysis of ALFF in VaMCI group showed that the temporal lobe and the Para hippocampal gyrus were closely related to memory, and that episodic memory was mainly impaired in early VaMCI period.

Compared with the NC group, there were 6 clusters with decreased ReHo values in VaMCI group, which were located in the left temporal lobe, occipital lobe, and left middle temporal gyrus, with statistical differences (*P* < 0.05). No statistical difference was noted in the left inferior temporal gyrus, left Para hippocampal gyrus, and insula (*P* > 0.05). There were 4 clusters with increased ReHo values, which were the left superior temporal gyrus, the right frontal lobe, the left frontal lobe, and the left inferior frontal gyrus (*P* < 0.05). Temporal lobe and occipital lobe are important brain areas involved in the subcortical circuits of the brain. The middle temporal gyrus is involved in cognitive and memory functions (31), and the occipital lobe is responsible for processing of language, motion sensation, and abstract concepts (32). Chen et al. used fMRI to study the ReHo value of VaMCI patients in resting state. The findings were consistent with the conclusions in this study (33), indicating that the spontaneous brain activity was reduced in these areas mentioned above, reflecting the overall cognitive impairment of patients to a certain extent. In a word, VaMCI patients have cognitive impairment and abnormally increased spontaneous brain activity, especially in the left parietal lobe and the right temporal lobe. At rest, VaMCI patients show decreased whole-brain ReHo in the left medial temporal lobe and occipital lobe. Hypertension is a high-risk factor for cognitive impairment in VaMCI patients. The study can provide a theoretical basis for early diagnosis of VaMCI.

## Conclusion

In the study, the fMRI was performed on subjects to identify the ALFF and Reho values and to analyze their association with VaMCI. It can be concluded that VaMCI patients have cognitive impairment and abnormally increased spontaneous brain activity, especially in the left parietal lobe and the right temporal lobe. At rest, VaMCI patients show decreased whole-brain ReHo in the left medial temporal lobe and occipital lobe. Hypertension is a high-risk factor for cognitive impairment in VaMCI patients. However, some limitations in the study should be noted. The sample size is small, which will reduce the power of the study. In the follow-up, an expanded sample size is necessary to strengthen the findings of the study. All in all, the study can provide a theoretical basis for early diagnosis of VaMCI.

## Data Availability Statement

The original contributions presented in the study are included in the article/supplementary material, further inquiries can be directed to the corresponding author/s.

## Ethics Statement

The studies involving human participants were reviewed and approved by Department of Neurology, Changzhi People's Hospital, Changzhi Medical College. The patients/participants provided their written informed consent to participate in this study. Written informed consent was obtained from the individual(s) for the publication of any potentially identifiable images or data included in this article.

## Author Contributions

LZ wrote the manuscript. YL, LB, and QL collected and analyzed general data of patients. XZ and BZ were responsible for Follow-up indexes analysis. BZ helped with statistical analysis. All authors read and approved the final manuscript.

## Funding

This work was supported by Scientific Research Project of Shanxi Provincial Health Commission in 2021 (No. 2021012). The key special project of “four batches” of science and technology innovation plan of Shanxi Provincial Health Commission (No. 2020XM38).

## Conflict of Interest

The authors declare that the research was conducted in the absence of any commercial or financial relationships that could be construed as a potential conflict of interest.

## Publisher's Note

All claims expressed in this article are solely those of the authors and do not necessarily represent those of their affiliated organizations, or those of the publisher, the editors and the reviewers. Any product that may be evaluated in this article, or claim that may be made by its manufacturer, is not guaranteed or endorsed by the publisher.
